# Editorial: Neural circuits revealed, volume II

**DOI:** 10.3389/fncir.2022.1023142

**Published:** 2022-09-21

**Authors:** Mariano Soiza-Reilly, Elizabeth Hanson Moss, Benjamin R. Arenkiel

**Affiliations:** ^1^Instituto de Fisiología, Biología Molecular y Neurociencias (IFIBYNE), Consejo Nacional de Investigaciones Científicas y Técnicas (CONICET), Universidad de Buenos Aires, Buenos Aires, Argentina; ^2^Department of Molecular and Human Genetics, Baylor College of Medicine, Houston, TX, United States

**Keywords:** viral tracing, optogenetics, ultrastructure (electron microscopy), connectome, electrophysiology, brain circuit mechanisms, behavior and cognition

The appropriate function of the nervous system relies on precise patterns of connectivity among hundreds to billions of neurons. Robust genetic blueprints give rise to complex neural circuits—conserved patterns of neural connectivity between morphologically and functionally diverse sets of neurons. Such circuits are ultimately responsible for the planning and execution of behaviors necessary for survival. Although it is well-established that individual neurons represent the elemental building blocks of the brain, the next frontier is understanding the functional architecture of neural circuits, and how they facilitate sensation, movement, cognition, and perception.

To unravel the details of nervous system function, we must consider not only the morphological and physiological properties of individual neurons, but also their connectivity patterns within and across brain regions. Advances in molecular genetic, viral engineering, and imaging technologies are fueling new insights into how interconnected neural networks function. With such tools in hand, we can better address long-standing, unanswered questions about structural and functional features of neural circuits with a remarkable level of specificity and precision.

This Research Topic: “*Neural circuits revealed, volume II*” revisits the original “*Neural circuits revealed*,” published in 2014, and highlights advances of the last decade in technologies for targeting, measuring, and manipulating increasingly precise neural circuits in a variety of models and conditions. A clear example of recent technological progress is depicted in the article by Morgan et al., in which the authors describe a novel methodological pipeline to integrate confocal fluorescence microscopy with the ultrastructural interrogation of neural circuits by electron microscopy. Their approach, termed multi-resolution correlated light and electron microscopy (mrCLEM), allows deep neural circuit structural characterization, and provides a foundation from which to investigate the functional features and implications of precisely-defined circuits.

For functional investigation, a broad and ever-expanding toolkit of viral and genetic techniques has evolved that now allows selective labeling and manipulation of individual neurons and their respective circuits. In their review, Hui et al. lay out the palette of different viral strategies that enable precise optogenetic and chemogenetic exploration of circuit function. Then, along these lines, Swanson et al. discuss state-of-the-art recombination-based and activity-driven genetic approaches to target, manipulate, and monitor the activity of specific brain cell populations.

Understanding how sensory inputs are encoded and then integrated with context, state, and reward information to inform behavioral output, represents a major challenge to current neuroscience. However, novel circuit interrogation methods are shedding light on these complex processes. In this article series, Moran et al. use *in vivo* two photon imaging of the glutamate sensor iGluSnFR to reveal inupt/output transformations, and glutamate-driven circuit modulation in the olfactory bulb during odor inhalation. Downstream of sensory input, subcortical midbrain structures interact with cortical circuits to process and integrate sensory information. An example of this circuit organization is found in the inferior colliculus, which integrates auditory signals for sound localization. Using retrograde tracing, Anair et al. describe a new commissural pathway that connects both sides of the inferior colliculus, engaging a GABAergic neuronal population that expresses neuropeptide Y. At the same time, reward-related and motivational processes influence the transformation of sensory input into behavioral output. In this system, prefrontal cortical regions communicate to local and long-range circuits within the ventral tegmental area (VTA), where dopamine transmission plays a central role in reward processing. Recent advances in the organizational features of VTA circuits and the interaction between dopamine and glutamatergic pathways are further discussed by Cai and Tong.

In addition to information processing in sensory and reward circuits, novel tools for targeting and interrogating circuits are also revealing mechanisms by which these circuits form in the developing brain, and how they change into adulthood. Ferrer and De Marco García describe when and how GABAergic circuits shape the assembly and function of developing sensory cortices, including an in-depth discussion of how intersectional genetics, imaging, and transcriptomics are being used to investigate highly-specific circuit components, and the genetic expression pathways that define them. Adding to this, Rupert and Shea review the latest findings on mechanisms by which GABAergic interneurons regulate plasticity in developing and adult brains, with a focus on the unique properties of cortical Parvalbumin-expressing interneurons that regulate sensory-driven plasticity in adulthood.

Together, this Research Topic brings to the readers novel, state-of-the-art methods, concepts, and perspectives about the structure and function of the brain. It revisits and builds upon the technology introduced in the original volume, as well as introduces new concepts and methods. As such, the collected articles and reviews provide an up-to-date overview of how newly-developed neurotechnologies are being applied to better enable highly precise interrogation of circuit architecture and function.

## Author contributions

All authors listed have made a substantial, direct, and intellectual contribution to the work and approved it for publication.

## Funding

MS-R is a researcher from the Consejo Nacional de Investigaciones Científicas y Técnicas (CONICET) of Argentina.

## Conflict of interest

The authors declare that the research was conducted in the absence of any commercial or financial relationships that could be construed as a potential conflict of interest.

## Publisher's note

All claims expressed in this article are solely those of the authors and do not necessarily represent those of their affiliated organizations, or those of the publisher, the editors and the reviewers. Any product that may be evaluated in this article, or claim that may be made by its manufacturer, is not guaranteed or endorsed by the publisher.

